# Quo Vadis LIPSS?—Recent and Future Trends on Laser-Induced Periodic Surface Structures

**DOI:** 10.3390/nano10101950

**Published:** 2020-09-30

**Authors:** Jörn Bonse

**Affiliations:** Bundesanstalt für Materialforschung und -prüfung (BAM), Unter den Eichen 87, D-12205 Berlin, Germany; joern.bonse@bam.de; Tel.: +49-30-8104-3562

**Keywords:** laser-induced periodic surface structures (LIPSS), ripples, applications, direct laser interference patterning (DLIP), surface functionalization, literature survey, review articles, special issues, scientific workshops, European funding strategies

## Abstract

Nanotechnology and lasers are among the most successful and active fields of research and technology that have boomed during the past two decades. Many improvements are based on the controlled manufacturing of nanostructures that enable tailored material functionalization for a wide range of industrial applications, electronics, medicine, etc., and have already found entry into our daily life. One appealing approach for manufacturing such nanostructures in a flexible, robust, rapid, and contactless one-step process is based on the generation of laser-induced periodic surface structures (LIPSS). This Perspective article analyzes the footprint of the research area of LIPSS on the basis of a detailed literature search, provides a brief overview on its current trends, describes the European funding strategies within the Horizon 2020 programme, and outlines promising future directions.

## 1. Introduction

During the last five decades, laser-induced periodic surface structures (LIPSS) have gained remarkable attention and developed into a scientific evergreen [1]. This is based on the simplicity and robustness of the single process step required for their manufacturing that can be performed in ambient air and that is fully compatible with industrial demands on costs, reliability, and productivity. Depending on the selected materials and specific irradiation conditions, the processing of LIPSS enables a large variety of various types of surface functionalization that become possible through different feature sizes, ranging between a few tens of nanometers up to several micrometers. This allows for addressing many applications in the fields of optics, electronics, fluidics, mechanical engineering, and medicine [2,3,4,5,6].

LIPSS are an arrangement of (quasi)periodic topographic lines representing a linear surface grating structure. They can be classified according to their spatial periods (Λ) and the orientation to the linear laser beam polarization used for their generation [1]. Many variants in naming the phenomenon of LIPSS can be found in the literature: some authors refer to ripples, or nanoripples, or even to near-wavelength or deep-subwavelength structures. Note that sometimes, other types of surface structures, such as hexagonally arranged nanometric protrusions or so-called micrometric Grooves or Spikes [7], are also referred to as LIPSS.

**Figure 1:**
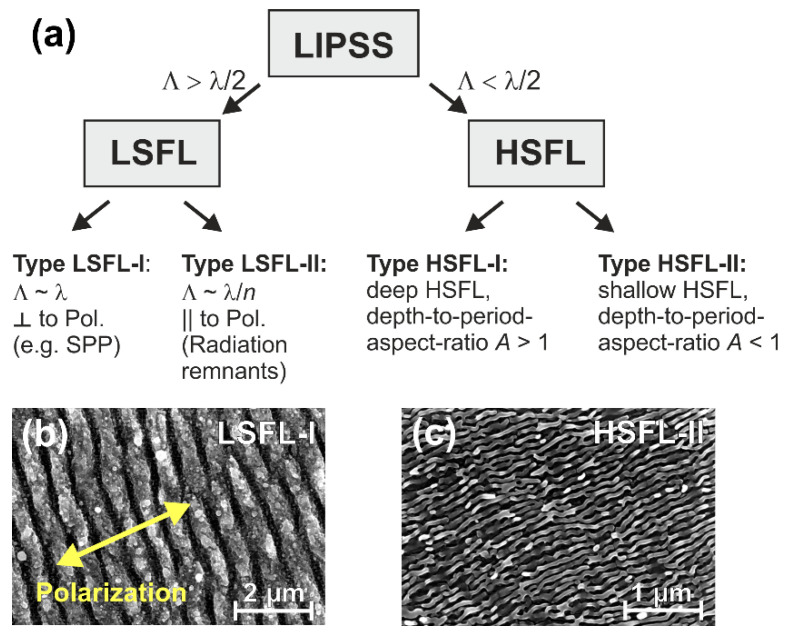
(**a**) General classification of laser-induced periodic surface structures (LIPSS) into different types of low spatial frequency LIPSS (LSFL) and high spatial frequency LIPSS (HSFL). © 2016 IEEE. Reprinted, with permission, from [1], Bonse, J.; Höhm, S.; Kirner, S.V.; Rosenfeld, A.; Krüger, J. Laser-induced periodic surface structures—A scientific evergreen, *IEEE J. Sel. Top. Quantum Electron,* **2017**, *23*, 9000615. SEM micrographs of near wavelength-sized LSFL-I (**b**) and sub-wavelength HSFL-II (**c**) on Ti6Al4V surfaces after irradiation with multiple fs-laser pulses in air (pulse duration τ = 30 fs, center wavelength λ = 790 nm, pulse repetition frequency 1 kHz). Note the different magnifications. The double arrows in (**b**) mark the direction of laser beam polarization. (**b**,**c**) are reproduced from Kirner et al. [9], Nanometer-resolved chemical analyses of femtosecond laser-induced periodic surface structures on titanium, *J. Appl. Phys,* **2017**, *122*, 104901, https://dx.doi.org/10.1063/1.4993128, with the permission of AIP Publishing.

Figure 1a provides the common classification of different types of grating-like LIPSS observed upon irradiation with ultrashort laser pulses. Low spatial frequency LIPSS (LSFL) have periods larger than half the laser irradiation wavelength (λ). They are either perpendicular (LSFL-I) or parallel (LSFL-II) to the laser beam polarization, depending on their specific formation mechanism [1,8]. An example of the LSFL-I type structures on the surface of Ti:sapphire femtosecond (fs-) laser-irradiated Ti6Al4V titanium alloy surface is provided in Figure 1b.

In contrast, high spatial frequency LIPSS (HSFL) exhibit periods smaller than λ/2 and may be formed either as rather deep surface gratings (HSFL-I, depth-to-period aspect ratio *A* > 1; predominantly observed on dielectrics and semiconductors) or as very shallow surface gratings (HSFL-II, depth-to-period aspect ratio *A* < 1; mainly observed on metals). Figure 1c exemplifies some HSFL-II structures formed for somewhat different irradiation conditions on the surface Ti6Al4V alloy.

Particularly, the last two decades provided remarkable new insights in the dynamics, the control, and the formation mechanisms of LIPSS. Currently, the LIPSS community is further exploring possible applications of these surface nanostructures. This Perspectives article does not aim to provide a complete scientific overview on LIPSS (for that, the reader is referred to available review articles) and it will not be able to consider all current research activities. It rather intends to discuss the research area of LIPSS from a personal researcher’s point of view, embedded in the European research environment, analyzing the current literature (Section 2) and highlighting some activities, projects, and recent trends which are expected to influence and foster the future developments in this field of research (Section 3).

## 2. Analysis of the Research Area of LIPSS

### 2.1. Available Literature

#### 2.1.1. Peer Reviewed Articles (1982–2020)

The current situation in the research area of LIPSS was analyzed through a literature search in the “ISI Web of Science—Core Collection” database, performed on 3 August 2020. In order to optimize the quality of the results, the precise search term “Laser-induced Periodic Surface Structures” was used in the category “Topic”, since the alternative term “Ripples” or other variants turned out to be too unspecific and generated too many incongruous hits. Based on this search strategy, 1111 publications were identified. The set of these publications is further analyzed in the following.

**Figure 2:**
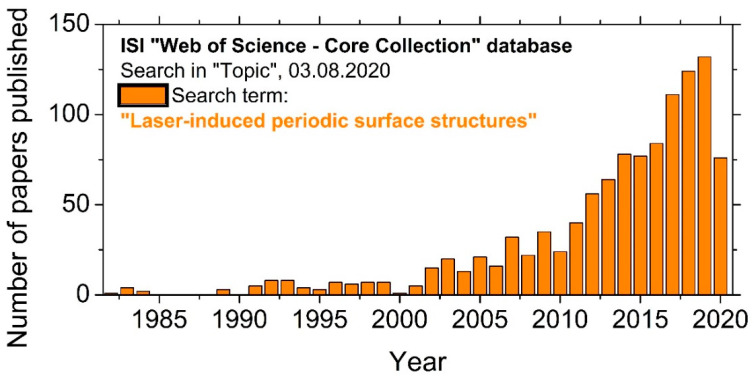
Research activities in the area of LIPSS, exemplified by the number of papers published per year—matching on 3 August 2020 in the “ISI Web of Science—Core Collection” database to the search term “Laser-induced Periodic Surface Structures”.

Figure 2 shows the number of annually published papers vs. the publication year since the term LIPSS was launched in the scientific literature by van Driel and co-workers in 1982 [10]. Already, during the first half of the 1980s, groundbreaking research was performed and published by two North American groups around van Driel and Sipe [10,11,12,13] in Canada, and around Fauchet and Siegman [14,15] in the USA. It included already time-resolved studies on the formation of LIPSS and material specific fundamental theories of these surface structures. That knowledge, based mainly on nanosecond (ns-) laser generated LIPSS, was summarized in some excellent review articles [16,17,18,19]. At that time, not too many questions were left open since the near-wavelength-sized LIPSS (LSFL) usually observed for ns-laser irradiation were successfully explained. Since industrially relevant applications were not developed, the academic interest on LIPSS dropped again, and during the 1990s, rather constant research activities with less ten paper published per year can be seen in Figure 2. That situation changed around the turn of the millennium, caused by the discovery of a new type of LIPSS with sub-wavelength characteristics (HSFL) that were observed upon irradiation with ultrashort laser pulse durations in the picosecond (ps-) to fs-range. This trend, visible in the rising publication rate, was supported by the increasing availability of ultrashort laser systems and is still ongoing with currently more than 130 publications per year.

**Figure 3:**
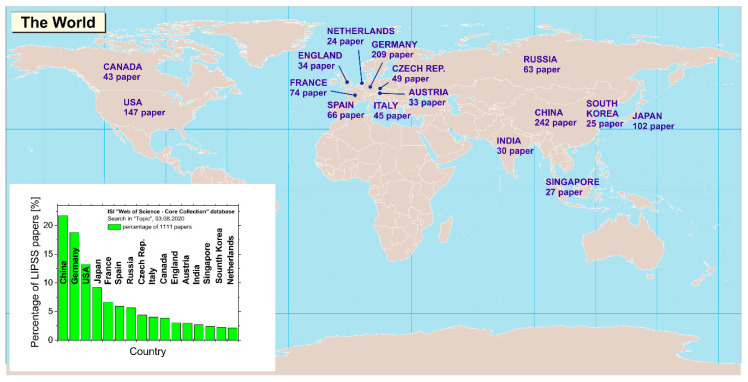
Geographic distribution of research on LIPSS, visualized via the assignment of the 16 countries having published most scientific articles in this field since 1982. The inset ranks the countries according to their percentage on the entire data set consisting of 1111 publications.

The same set of data was analyzed with regard to geographical origins of the 1111 publications. Figure 3 visualizes, in a map of the world, the sixteen countries being most active in LIPSS research along with the associated absolute number of the publications originating from them. The inset in the lower left corner orders these countries according to the corresponding percentage when relating the absolute number of publications to the total number in the data set. Note that the sum of percentages (number of papers) exceeds 100% (1111) since a publication may arise from international collaborations of research groups assigned to different countries.

The largest number of articles was published from groups from China (242), followed by Germany (209), the USA (147), Japan (102), France (74), Spain (66), Russia (63), Czech Republic (49), Italy (45), Canada (43), England (34), Austria (33), India (30), Singapore (27), South Korea (25), and the Netherlands (24). While early research on LIPSS was mainly performed in North America, currently, the most intense activities are located in Europe, China, Japan, and Russia.

Table 1 lists the research institutions publishing most actively in the field of LIPSS, ordered by the number of publications and the corresponding percentages. The top three (and, in total, five out of the top ten institutions) represent national research academies/councils that confine publications of multiple different groups working on LIPSS.

The top 10 scientific journals and corresponding publishers with articles in the area of LIPSS are provided in Table 2, ordered by the number of publications and the corresponding percentages. Most publications appeared in the journal *Applied Surface Science* (167, 15%), followed by the *Proceedings of SPIE* (95, 8.6%), *Applied Physics A* (69, 6.2%), *Journal of Applied Physics* (48, 4.3%), *Applied Physics Letters* (40, 3.6%), *Optics Express* (40, 3.6%), *Journal of Laser Micro/Nanoengineering* (25, 2.3%), and three other journals with less than 2% contained in the analyzed data set. According to the scope of these journals, the publications are interdisciplinary and mainly associated with research areas of physics, material science, optics, chemistry, technology, and engineering.

#### 2.1.2. Review Articles

The state of knowledge on LIPSS up to the middle of the 1980s was reviewed by different groups [16,17,18]. The renewed and increased attention to LIPSS at the turn of the millennium has led to numerous new experimental findings featuring the capabilities of ultrashort laser pulses, i.e., enabling a non-equilibrium between the electronic system and the lattice system of irradiated solids. Through tailored energy deposition via polarization-controlled double-fs-pulse sequences [20], or in time-resolved coherent scattering [21,22], diffraction [23] and microscopy [24] experiments, important new insights were gained on the early stage of LIPSS formation, particularly on the relevance and involvement of surface plasmon polaritons (SPPs)—for details, see [1].

Other review articles [25,26] discussed the similarity and relation between the HSFL and some volumetric nanostructures observed upon focusing a fs-laser beam tightly into the bulk of transparent dielectrics [27]. The direct link between both types of structure could finally be provided though time-resolved 3D finite-difference time-domain (FDTD) numerical simulations, identifying the non-radiative near-field scattering at nanoscopic defects as shared origin [8].

Very recently, the competition between electromagnetic models and matter reorganization theories was reviewed [28]: in view of 3D-FDTD simulations combined with a two-temperature model, the equation-of-state, and the Navier–Stokes equations [29], it can be stated that two classes of theories (electromagnetics vs. hydrodynamics) currently merge into a joint view on LIPSS, allowing to consider both the early electromagnetic excitation and energy deposition and the following matter reorganization stages.

A still-ongoing and very active trend in the research of LIPSS is the study of their applications for various surface functionalizations since LIPSS can modify the optical, mechanical, and chemical properties of irradiated surfaces for industrial applications in optics, tribology, medicine, etc. Up-to-date surveys on this topic are found in recent reviews articles [2,3,4,5]. Many applications are particularly inspired by nature and enable surface engineering through tailored biomimetic laser-generated surface structures, reviewed in [6,30].

#### 2.1.3. Special Issues

Various peer-reviewed scientific journals have recognized the potential of the topic and organized Special Issues related to LIPSS. Table 3 lists a selection that is ordered chronologically. Most of them are currently arranged by the publisher MDPI that is even running several topically overlapping issues at the same time.

### 2.2. Scientific Conferences and Workshops

The most important conferences in the field of laser–matter interaction nowadays all organize individual sessions devoted to LIPSS. The most relevant ones are the biannual International Conference on Laser Ablation (COLA), the annual Spring Meeting of the European Materials Research Society (E-MRS), the annual International Symposium on Laser Precision Microfabrication (LPM), several symposia at the annual SPIE Photonics West Conference, the annual Conference on Lasers and Electro-Optics (CLEO^®^), the International High Power Laser Ablation Symposium (HPLA), the annual International Conference on Advanced Laser Technologies (ALT), the triannual International Symposium “Fundamentals of Laser Assisted Micro- and Nanotechnologies” (FLAMN), the biannual International Conference on Photo-Excited Processes and Applications (ICPEPA), and the annual industry near International Congress on Applications of Lasers and Electro-Optics (ICALEO^®^), among some other smaller conferences.

Since approximately ten years ago, a highly specialized, invitation-based International Workshop on Laser-Induced Periodic Surface Structures (LIPSS) has been organized by Europe’s LIPSS community, typically with only 20–40 attendees to keep the format informal, allowing to stimulate in-depth discussions. It was initiated in 2011 by the University of Twente (The Netherlands) and later organized in Germany, Czech Republic, France, Greece, and Slovenia. Table 4 recalls its history until today.

In 2017, an additional public workshop, Laser Processing for Bionic Applications, was organized at BAM for the dissemination of a European research project (LiNaBioFluid, see Section 3.5) to an international industrial and academic audience.

## 3. Recent (Ongoing) Trends

This section elucidates some recent trends in the field of LIPSS, discussing current theoretical and surface analytical developments, a comparison to alternative surface processing techniques, most recent applications, and a view on the European research landscape of LIPSS.

### 3.1. Electromagnetics vs. Matter Reorganization

During the past two decades, there was a vivid and stimulating discussion regarding the origin of LIPSS, particularly whether these structures are caused via ultrafast energy deposition mechanisms that are acting during the absorption of optical radiation, or via matter reorganization effects that are occurring after the irradiation process [1]. Taking advantage of the ultrashort pulse duration available in the fs- to ps-range, time-resolved approaches based on coherent scattering [21], diffraction [23], microscopy [24], and polarization-controlled multi-wavelength double-fs-laser pulse irradiation [20] have experimentally proven about ten years ago that the ultrafast excitation stage is essential for the formation of LIPSS upon ultrashort pulse laser irradiation. Recently, however, important improvements were made regarding the theoretical modeling, particularly via electromagnetic FDTD, [31,32] and hydrodynamic [29,33] and molecular dynamics (MD) simulations [34,35] considering post-irradiation matter reorganization. Currently, both theoretical approaches, i.e., electromagnetics and matter reorganization, are merging into a joint view where—depending on the irradiation conditions and materials—specific electromagnetic/reorganization aspects can dominate. A detailed review is provided in [28].

### 3.2. Topography vs. Chemistry

There is an ongoing and vivid debate on the relevance of chemical effects accompanying the formation of LIPSS. It is triggered by the fact that many applications of LIPSS, e.g., for surface wetting control, cell and bacterial adhesion, or the management of friction and wear, are affected by both the sample topography and the local surface chemistry. While most of the early research focused mainly on topographical effects, currently, the influence of the surface chemistry is increasingly investigated [9,36,37,38,39], studying, in detail, superficial oxidation upon irradiation in air environment and post-irradiation molecular adsorption phenomena.

Depth-resolved surface analytical studies on LIPSS based on Auger electron microscopy (AEM) [9], time-of-flight secondary ion mass spectrometry (TOF-SIMS) [40], and glow-discharge optical emission spectroscopy (GD-OES) [41] indicate a graded oxidized surface layer of several hundreds of nanometer thickness. Such nanostructured oxide layers may, however, exhibit beneficial tribological effects, particularly in combination with “anti-wear” additives, such as zinc-dialkyl-dithiophosphate (ZDDP), contained in lubricants, such as commercial engine oil [41,42,43].

The impact of laser processing on surface wetting behavior became more clear already ten years ago through a study of Kietzig et al. [44], reporting that metal surfaces are superhydrophilic (water contact angles ~ 0°) right after laser irradiation of different types of surface structures (LIPSS, Grooves, Spikes) due to laser-induced oxidation. On the timescale of several days or even longer, and without any topographic changes, the surfaces turn nearly superhydrophobic (water contact angles > 150°) due to the adsorption of hydrocarbon molecules or other contaminants from the ambient environment [44,45,46]. Since the presence of adsorbed molecules or contaminants at the surface is not necessarily stable against the storage conditions, surface cleaning, heat treatment [46], and other ageing effects, reliable industrial applications of these structures are difficult and may require additional stabilization steps.

### 3.3. LIPSS vs. DLIP

**Figure 4:**
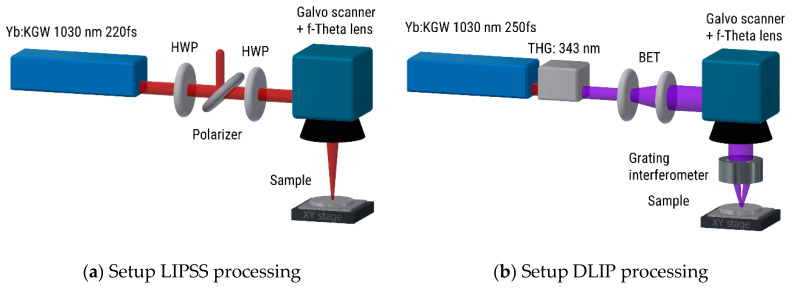
Different approaches for generating grating-like periodic surface structures. (**a**) Setup for processing of LIPSS. (**b**) Setup for direct laser interference patterning (DLIP) processing. Abbreviations: HWP: half-wave plate; THG: third harmonic generator; BET: beam expanding telescope; reprinted from Rung et al. [47], Possibilities of Dry and Lubricated Friction Modification Enabled by Different Ultrashort Laser-Based Surface Structuring Methods, *Lubricants,* **2019**, 7, 43. Copyright 2019 under Creative Commons BY 4.0 license. Retrieved from https://doi.org/10.3390/lubricants7050043.

Another method for producing line grating like periodic surface structures relies on direct laser interference patterning (DLIP) by superimposing two focused laser beams at the sample surface. Figure 4 compares the processing of LIPSS by using a Galvanometer scanner and an f-Theta lens (Figure 4a) with the more complex DLIP setup (Figure 4b), where an additional grating interferometer is employed to realize the two-beam interference here [47].

**Figure 5:**
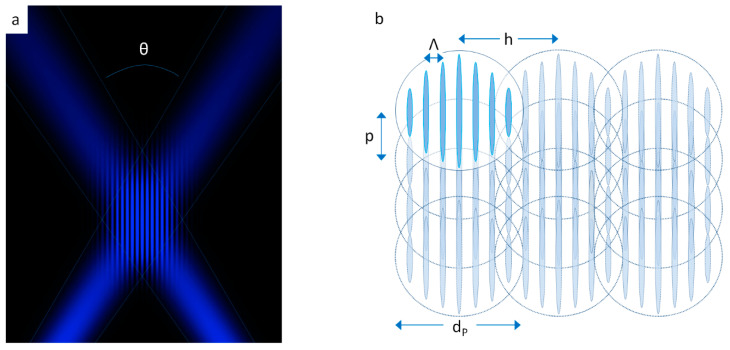
(**a**) DLIP interference pattern generated by two laser beams overlapping at an angle *θ*. (**b**) Scanning processing by displacement of multiple DLIP pixels. *p*: pulse (spot) separation, *h*: hatch distance; *d*_p_: DLIP pixel size; Λ: DLIP spatial period. The direction of scanning is vertical here. Reprinted from Mezera et al. [48], Hierarchical Micro-/Nano-Structures on Polycarbonate via UV Pulsed Laser Processing, *Nanomaterials*, **2020**, 10, 1184. Copyright 2020 under Creative Commons BY 4.0 license. Retrieved from https://doi.org/10.3390/nano10061184.

When choosing proper laser pulse energies for DLIP, material removal (ablation) is caused selectively at the interference maxima of the spatially modulated intensity distribution generated by the two incident laser beams visualized in Figure 5a. The spatial period (Λ_DLIP_) of the interference pattern can be controlled by the laser wavelength (λ) and the angle of incidence between the two interfering laser beams (*θ*) via Λ_DLIP_ = λ/[2sin(*θ*/2)], confined in micrometer-sized DLIP pixels that can be individually addressed and scanned across the surface (see Figure 5b). The minimum spatial period of the interference pattern is then given by Λ_DLIP_ = λ/2. This restriction imposed by the optical diffraction limit can be overcome for LIPSS when HSFL structures are processed. The latter can exhibit spatial periods of only some tenths of the laser wavelength [1,5]. LSFL spatial periods, however, typically range between λ and λ/2 [1,5].

**Figure 6:**
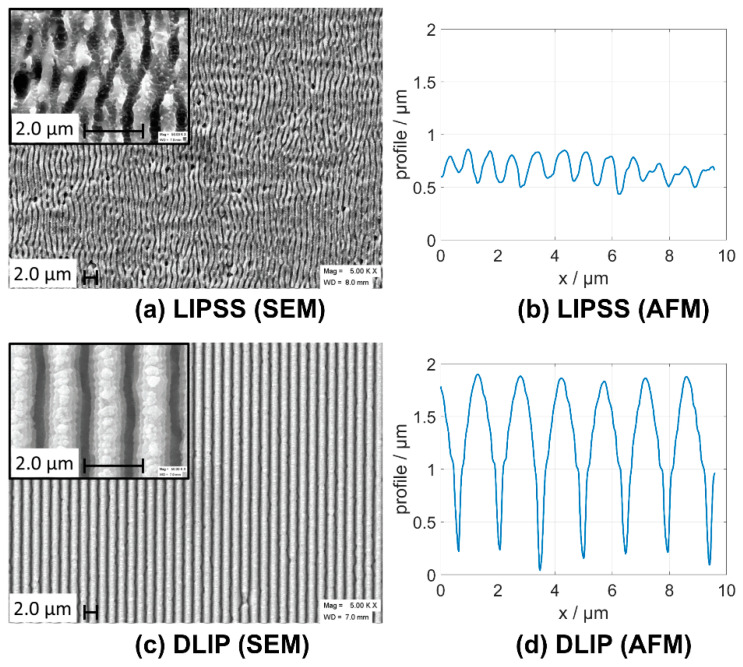
Laser-generated periodic surface structures on steel using the LIPSS and DLIP methods. The scanning electron microscopy (SEM) micrograph (**a**) indicates quasi-periodic LIPSS (LSFL). The corresponding atomic force microscopy (AFM) cross-section (**b**) indicates a depth modulation profile. Structures processed by DLIP reveal a larger regularity in the SEM micrograph (**c**) and larger depth modulations in the AFM cross-section (**d**). Adapted from Rung et al. [47], Possibilities of Dry and Lubricated Friction Modification Enabled by Different Ultrashort Laser-Based Surface Structuring Methods, *Lubricants*, **2019**, 7, 43. Copyright 2019 under Creative Commons BY 4.0 license. Retrieved from https://doi.org/10.3390/lubricants7050043.

The depth of the DLIP ablation pattern can be independently controlled from its period by a proper choice of the laser pulse energy (fluence) and the number of pulses applied per pixel. In contrast, for LIPSS processing, the spatial period and the depth of the LIPSS also crucially depend on the fluence and the number of pulses per spot area, but both parameters usually cannot be controlled independently. For LIPSS, the surface modulation depth is typically limited at ~400 nm for LSFL and ~1000 nm for HSFL (dielectrics), depending on the materials and formation mechanisms. DLIP structures can have significantly larger modulation depths and higher regularities of periodic surface patterns, as demonstrated in Figure 6. The larger modulation depths may be beneficial for the tribological performance of the laser-structured surfaces [47] as they allow to better confine some lubricant in the tribological contact area [49].

**Figure 7:**
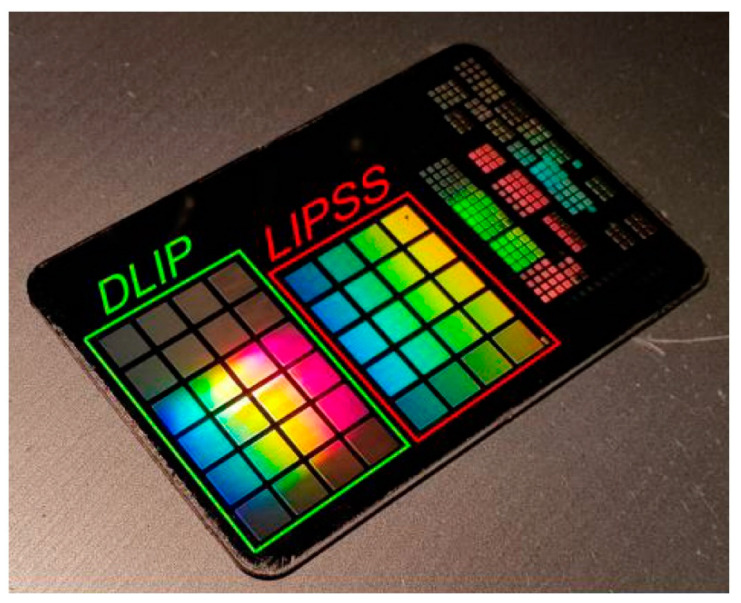
Photograph of structural colors generated on a polished steel plate by DLIP and LIPSS processing, as indicated on the sample. Reproduced from Soldera et al. [50], with permission from Japan Laser Processing Society.

The differences in the topographic characteristics also affect the optical properties of the (quasi-)periodic surface structures that may be used for safety tags, information encoding, and decoration purposes. The latter is based on the fact that surface grating structures can cause spectral and angular dependent diffraction of light, resulting in colorization effects of the treated samples. Figure 7 shows a photograph of a steel plate that was textured with similar grating-like DLIP structures and LIPSS (spatial periods ~1 µm) upon illumination with a point-like white light source [50]. The LIPSS patterned surface areas show a homogeneous colorization and a rather matte appearance, while the DLIP-treated regions exhibit brighter colors and a glossy appearance. Furthermore, the angular spectrum of the diffracted light is narrower in the case of the DLIP gratings when compared to the LIPSS. All these aspects finally point back to the different regularity of the surface structures, including deviations from the perfect grating geometry along with differences in their range of spatial periods.

Employing up-to-date laser and beam scanning technology, the LIPSS processing approach and the DLIP technique both can fulfill current industrial demands by processing patterns at processing rates approaching the m^2^/min level [51,52,53,54] Both surface patterning methods strongly depend on the coherence of the laser radiation used. The constraints, however, are somewhat different: while the contrast of the interference pattern generated in the DLIP pixel relies on global coherence (spatial and temporal), in LIPSS processing, only a local coherence of the beam is required, coupling the laser radiation to the material via scattering/interference at microscopic surface defects (roughness, absorption centers, etc.). Moreover, given the inherently large spectral bandwidth of ultrashort laser pulses and its impact on the resulting interference patterns, the DLIP technique is usually limited to pulse durations in the ps-range or longer. Table 5 compiles a direct comparison of surface texturing by DLIP or by LIPSS, summarizing the discussed aspects.

Recently, some groups started to investigate the hybrid processing of hierarchical surface structures of micrometric DLIP structures superimposed with nanometric LIPSS [48,55,56], which can combine the benefits of both approaches on the costs of an additional processing step.

### 3.4. Exploration of Other Applications

Other applications and of LIPSS that are currently explored are:

- Biomimetic surfaces: Nature provides many highly optimized surface functionalities that may be transferred to technical applications via tailored laser-processing, including LIPSS. Examples are dirt-repellent surfaces through the well-known lotus effect, anti-icing [57,58], the directional transport of liquids inspired by moisture-harvesting lizards [59] and bark bugs [60], antiadhesive surfaces inspired by cribellating spiders [61], or antibacterial [62,63,64,65,66,67], cell-repellent [68], and cell-stimulating/-adapting surfaces [69,70,71] for medical applications [72]. A detailed review of the laser engineering of biomimetic surfaces is provided in [6].
- Combined processing strategies: Currently, several research groups are exploring the combination of LIPSS with additional surface treatment techniques—either “in situ” during the laser processing, or “ex situ” after the laser-processing. Examples are: (i) combined laser processing strategies (such as in situ double-pulse treatments [20,73,74] or ex situ LIPSS + DLIP, see Section 3.3), or a two-step laser processing of microstructures (e.g., lines, grids, or more complex microfluidic channels) patterned additionally with nanostructures (LIPSS) [59,75]; (ii) the combination of LIPSS processing with thermal heat during [76,77] or after [78,79] laser irradiation; (iii) electrochemical post-processing, such as anodization [67,80]; or (iv) ion beam post-processing for altering the electrical conductivity [81].
- Improved regularity of LIPSS through surface overlayers: On dielectrics, the generation of large surface areas covered homogeneously with LIPSS is often very difficult when the single photon energy is significantly smaller than the band gap energy, i.e., when nonlinear absorption is required to couple the laser beam energy with the solid. Apart from the strategy to reduce the nonlinearity via the irradiation wavelength [82], another way to overcome this difficulty can lie in adding a very thin strongly-absorbing surface overlayer on the dielectric in order to facilitate resonant coupling effects of the laser radiation to the material underneath. For hexagonally arranged ablative nanobumps on glass, tens of nanometer thick copper and silver coatings were shown to be suitable [83,84]. Later, Kunz et al. demonstrated that large surface areas homogeneously covered by HSFL can be processed on fused silica by the help of an additional 20 nm-thick gold layer [85].
- LIPSS on thin films: Often, the selective structuring of thin film coatings is necessary for creating specific surface functionalities. Conventional surface structuring techniques are, however, often limited by small film thicknesses in the sub-micrometer range and high hardness or brittleness of the film materials. Hence, several groups are exploring the (contactless) formation of LIPSS on various overlayer materials [86,87]. Furthermore, following the general trend of research on graphene (triggered by the Nobel prize awarded in 2010), several authors studied the formation of LIPSS on graphene or graphene oxide-covered substrates [88,89,90,91,92]. It was demonstrated that LIPSS manifesting via structural modifications of the graphene or the material underneath can be used as local probe of plasmonic resonances [91,92].
- LIPSS for sensing applications: One of the first applications of LIPSS came up in the context of black silicon that can be generated upon ultrashort laser processing of silicon as hierarchical surface morphology consisting of micrometric Spikes [93] covered with nanometric LIPSS. It was recognized by Mazur and his co-workers at Harvard University (USA) that these surface structures can be used for building silicon-based photodetector devices with an enhanced optical sensitivity in the (near) infrared spectral region. Later, this idea was commercialized and is already being used for night vision cameras [94]. Another sensing application of LIPSS used in chemical analytics is based on surface-enhanced Raman spectroscopy (SERS). The effect is based on electromagnetic near-field enhancement in the vicinity of very sharp surface topographic features and may be further enlarged by resonant effects, such as the excitation of SPPs. It was demonstrated that the SERS effect on LIPSS on polymers that were overcoated with gold can increase the detection sensitivity of specific analyte molecules by several orders of magnitude [95,96]. Additionally, the localized laser surface processing could help to spatially confine the analyte solution during an additional evaporation-based concentration enhancement step [97,98].
- Magnetic and superconducting properties of LIPSS: Several authors started to investigate the impact of LIPSS on magnetic [99,100,101] and superconducting properties [102].

### 3.5. Funding Stratgies for LIPSS: The European H2020 Perspective

The European Commission (EC) supports the research on LIPSS currently via their Horizon 2020 (H2020) programme. Table 6 lists some corresponding projects that are funded through different schemes. Those H2020 funding opportunities are briefly discussed in the following section.

Laserlab-Europe is a consortium of currently ~30 major laser research infrastructures, located in 18 European countries (https://www.laserlab-europe.eu). The network has been in development since 2004 and provides access to their laser facilities to a broad user community, pursues research and development for improved access and research opportunities, and aims to foster networking activities for strengthening the European laser research landscape. Currently, it is supported by the EC in the Integrating Activities for Advanced Communities programme, as a part of the Research and Innovation Action (RIA) scheme.

Within the same RIA scheme, EC also supports Future and Emerging Technologies (FET), e.g., in the frame of FET Open projects (e.g., LiNaBioFluid, BioCombs4Nanofibers), FET Proactive projects (e.g., LaserImplant), or smaller Coordination and Support Actions (e.g., CellFreeImplant, LaBionicS, BioProMarL). Such interdisciplinary projects develop early-stage science and technology research exploring new foundations for radically new future technologies and should provide a long-term vision of high-risk research.

Other LIPSS projects are supported within the RIA scheme by the Industrial Leadership programme (e.g., TresClean, LAMPAS, FemtoSurf) or the Technologies for Factories of the Future programme (e.g., LASER4SURF). Fostering new skills by means of excellent initial training of early-stage researchers is provided through Marie Skłodowska-Curie Innovative Training Networks (e.g., Laser4Fun).

In 2019, the EC’s European Innovation Council Pathfinder started an initiative for the dissemination and exploitation of results obtained within FET projects through the Future Tech Week (http://futuretechweek.fetfx.eu/).

## 4. Future Trends and Open Questions

Through ultrafast time-resolved experiments and the development of advanced numerical simulations combining electromagnetic and matter reorganization theories, a unified view on LIPSS is currently being developed. While the role of the electromagnetic scattering in LIPSS formation is already widely clarified, there is still an ongoing debate on the matter reorganization side, whether the Marangoni instability or the Rayleigh–Taylor instability provide dominant contributions in the hydrodynamic relaxation stage that is following the optical material excitation.

Moreover, an ongoing interest in LIPSS for practical applications is observed, e.g., for developing tailored surface functionalities. Current areal LIPSS processing rates are at the m^2^ min^−1^ level. It can be expected that future developments in laser technology enabling pulse repetition frequencies in the GHz range and modern laser scanner technology, along with smart scanning strategies for managing and optimizing the residual thermal load imposed to the laser irradiated material, will allow industrial processing rates at the m^2^ s^−1^ level. This will also further reduce the costs of LIPSS processing that were estimated to be ~0.05 €/cm^2^ in 2019 [3].

While most of the research on LIPSS was driven and interpreted on the basis of physical effects and models, it becomes clear, in practice, that additional effects involving the local surface chemistry and the environment must be considered and properly controlled for industrial or medical applications, e.g., for surface wetting, adhesion, implant functionalization, etc. It can be expected that the role of chemical effects accompanying the formation of LIPSS will represent an important future trend of research on LIPSS. The combination of MD and FDTD simulations may allow new future insights here. Moreover, in the context of numerical simulations, machine learning algorithms can enable the predictive modeling of optimized laser processing parameters for the desired surface topography [103].

Another future trend will be in the continued exploration of already-established and new surface functionalities that can be created through LIPSS. One particular aspect may be the long-term stabilization of the surface wetting characteristics (e.g., hydrophobicity or -philicity) of LIPSS in real-life environments and in daily use. In this context, the author expects that the remarkable number of research projects funded by the European Commission (see Section 3.5) will result in new and improved industrial applications in fields of mechanical engineering, healthcare, aviation, shipping, jewelry, or for consumer goods.

## Figures and Tables

**Table 1:** Top 10 international research institutions with publications associated to the scientific topic of LIPSS, evaluated on basis of the “ISI Web of Science” database ^1^.

Institution	Country	Number of Papers ^1^	Percentage ^1^
Centre National de la Recherche Scientifique, CNRS	FR	60	5.4%
Chinese Academy of Sciences	CN	54	4.9%
Consejo Superior de Investigaciones Científicas, CSIC	ES	53	4.8%
Bundesanstalt für Materialforschung und -prüfung, BAM	DE	49	4.4%
Max-Born-Institut für Nichtlineare Optik und Kurzzeitspektroskopie, MBI	DE	40	3.6%
Russian Academy of Sciences, RAS	RU	36	3.2%
University of Rochester	USA	34	3.1%
Czech Academy of Sciences, CAS	CZ	31	2.8%
CNRS Institute for Engineering Systems Sciences, INSIS	FR	30	2.7%
Université Jean Monnet Saint-Étienne	FR	30	2.7%

^1^ Search in “ISI Web of Science—Core Collection” database, 3 August 2020, search term “Laser-induced Periodic Surface Structures” in “Topic”, identifying a set of 1111 publications.

**Table 2:** Top 10 scientific journals and publishers with articles associated to the scientific topic LIPSS, evaluated on basis of the “ISI Web of Science” database ^1^.

Journal	Publisher	Number of Papers ^1^	Percentage ^1^
Applied Surface Science	Elsevier	167	15.0%
Proceedings of SPIE	SPIE ^2^	95	8.6%
Applied Physics A	Springer Nature	69	6.2%
Journal of Applied Physics	AIP ^3^	48	4.3%
Applied Physics Letters	AIP ^3^	40	3.6%
Optics Express	OSA ^4^	40	3.6%
Journal of Laser Micro/Nanoengineering	JLPS ^5^	25	2.3%
Physical Review B	APS ^6^	19	1.7%
Optics and Laser Technology	Elsevier	18	1.6%
Optics Letters	OSA ^4^	18	1.6%

^1^ Search in “ISI Web of Science—Core Collection” database, 3 August 2020, Search term “Laser-induced Periodic Surface Structures” in “Topic”, identifying a set of 1111 publications. ^2^ Society of Photo-Optical Instrumentation Engineers. ^3^ American Institute of Physics. ^4^ Optical Society of America. ^5^ Japan Laser Processing Society. ^6^ American Physical Society.

**Table 3:** Special issues related to LIPSS published in peer-reviewed scientific journals.

Journal	Publisher	Special Issue	**Date**
Journal of Laser Applications	LIA ^1^	“Generation of sub-100 nm Structures by Nonlinear Laser Material Interaction”	2012
Optical Materials Express	OSA ^2^	“Ultrafast Laser Modification of Materials (ULM)”	2013
MRS Bulletin	MRS ^3^	“Ultrafast Laser Synthesis and Processing of Materials”	2016
Nanomaterials	MDPI ^4^	“Laser-Based Nano Fabrication and Nano Lithography”	2018
Opto-Electronic Advances	IOE-CAS ^5^	“IAPLE Special Issue of Opto-Electronic Advances”	2019
Optical Materials Express	OSA ^2^	“Laser Writing”	2019
Lubricants	MDPI ^4^	“Laser-Induced Periodic Surface Nano- and Microstructures for Tribological Applications”	2020
Advanced Optical Technologies	De Gruyter	“Laser Micro- and Nano-Material Processing”	2020
Nanomaterials	MDPI ^4^	“Laser-Generated Periodic Nanostructures”	2020
Nanomaterials	MDPI ^4^	“Laser Synthesis and Modification of Materials at the Nanoscale”	2020
Nanomaterials	MDPI ^4^	“Laser Printing of Nanophotonic Structures”	2020
Photonics	MDPI ^4^	“Femtosecond Laser Micro/Nanofabrication”	2020
Nanomaterials	MDPI ^4^	“Laser Surface Functionalization on Nanomaterials”	2021
Nanomaterials	MDPI ^4^	“Nanopatterning of Bionic Materials”	2021

^1^ Laser Institute of America. ^2^ Optical Society of America. ^3^ Materials Research Society. ^4^ Multidisciplinary Digital Publishing Institute. ^5^ Institute of Optics and Electronics, Chinese Academy of Sciences.

**Table 4:** History of the International Workshop on Laser-Induced Periodic Surface Structures (LIPSS).

Year	Date	Host	Country
2011	10–11 October	University of Twente	NL
2012	3–4 October	Brandenburgische Technische Universität Cottbus	DE
2013	7 November	Bundesanstalt für Materialforschung und -prüfung (BAM), Berlin	DE
2014	11 November	HiLASE Centre, Dolní Břežany	CZ
2015	5 December	Laboratoire Hubert Curien, Saint-Étienne	FR
2016	24–25 November	Foundation of Research and Technology Hellas (FORTH), Heraklion	GR
2017	28–29 September	Brandenburgische Technische Universität Cottbus	DE
2018	27–28 September	Ruhr-Universität Bochum	DE
2019	26–27 September	University of Ljubljana	SL
2020/2021	To be decided	CNRS/Université d’Orléans	FR

**Table 5:** Comparison of single beam surface processing of LIPSS with two-beam based DLIP structures.

Property	DLIP (Two Beams)	LIPSS (One Beam)
Spatial period	>λ/2 ^1^	~λ/10 (HSFL)–~λ (LSFL)
Modulation depth [nm]	0–2000 [47]	<1000 (HSFL) [5]
		<400 (LSFL) [47]
Regularity of grating	++ ^2^	+ ^3^/o ^4^
Flexibility of processing	pixelwise during scanning	continuous scanning
Control of periods/depths	++ ^2^, independent	o ^4^, dependent
Complexity of setup	++ ^2^/+ ^3^	– ^5^
Areal processing rate (current state)	<m^2^/min	<1.5 m^2^/min [54]
Required beam coherence	global	local
Pulse duration	ps-cw	fs-cw

^1^ λ: laser irradiation wavelength. ^2^ ++: very good/very high. ^3^ +: good/high. ^4^ o: medium. ^5^ –: low.

**Table 6:** Research projects on LIPSS funded by the European Commission (EC) within the Horizon 2020 (H2020) programme.

Acronym	Name	Duration	Website
LASERLAB-EUROPE	The Integrated Initiative of European Laser Research Infrastructures	2019–2023	https://cordis.europa.eu/project/id/871124
LiNaBioFluid	Laser-induced Nanostructures as Biomimetic Model of Fluid Transport in the Integument of Animals	2015–2018	https://cordis.europa.eu/project/id/665337
Laser4Fun	European ESRs ^1^ Network on short pulsed laser Micro/Nanostructuring of Surfaces	2015–2019	https://cordis.europa.eu/project/id/675063/
TresClean	High throughput laser texturing of self-cleaning and antibacterial surfaces	2016–2020	https://cordis.europa.eu/project/id/687613/
LASER4SURF	Laser for mass production of functionalized metallic surfaces	2017–2021	https://cordis.europa.eu/project/id/768636
CellFreeImplant	Cell-free Ti-based Medical Implants due to Laser-induced Microstructures	2018–2019	https://cordis.europa.eu/project/id/800832/
LaBionicS	Laser Bionic Surfaces	2018–2020	https://cordis.europa.eu/project/id/801250/
LAMPAS	High throughput Laser structuring with Multiscale Periodic feature sizes for Advanced Surface Functionalities	2019–2021	https://cordis.europa.eu/project/id/825132/
FemtoSurf	Functional surface treatments using ultra-short pulse laser system	2019–2021	https://cordis.europa.eu/project/id/825512/
BioProMarL	Bio-inspired Protection of Marble with Lasers	2019–2021	https://cordis.europa.eu/project/id/852048/
BioCombs4Nanofibers	Antiadhesive Bionic Combs for Handling of Nanofibers	2019–2022	https://cordis.europa.eu/project/id/862016/
LaserImplant	Laser-induced hierarchical micro-/nano-structures for controlled cell adhesion at implants	2021–2022	https://cordis.europa.eu/project/id/951730

^1^ Early stage researchers.
